# Frequency-Oriented Transformer for Remote Sensing Image Dehazing

**DOI:** 10.3390/s24123972

**Published:** 2024-06-19

**Authors:** Yaoqing Zhang, Xin He, Chunxia Zhan, Junjie Li

**Affiliations:** 1 School of Basic Sciences for Aviation, Naval Aviation University, Yantai 264001, China; abbychn@126.com (Y.Z.); hexin6770@163.com (X.H.); zcxsun102324@163.com (C.Z.); 2 School of Electromechanical and Automotive Engineering, Yantai University, Yantai 264005, China

**Keywords:** remote sensing image dehazing, image restoration, frequency domain, fast Fourier transform

## Abstract

Remote sensing images are inevitably affected by the degradation of haze with complex appearance and non-uniform distribution, which remarkably affects the effectiveness of downstream remote sensing visual tasks. However, most current methods principally operate in the original pixel space of the image, which hinders the exploration of the frequency characteristics of remote sensing images, resulting in these models failing to fully exploit their representation ability to produce high-quality images. This paper proposes a frequency-oriented remote sensing dehazing Transformer named FOTformer, to explore information in the frequency domain to eliminate disturbances caused by haze in remote sensing images. It contains three components. Specifically, we developed a frequency-prompt attention evaluator to estimate the self-correlation of features in the frequency domain rather than the spatial domain, improving the image restoration performance. We propose a content reconstruction feed-forward network that captures information between different scales in features and integrates and processes global frequency domain information and local multi-scale spatial information in Fourier space to reconstruct the global content under the guidance of the amplitude spectrum. We designed a spatial-frequency aggregation block to exchange and fuse features from the frequency domain and spatial domain of the encoder and decoder to facilitate the propagation of features from the encoder stream to the decoder and alleviate the problem of information loss in the network. The experimental results show that the FOTformer achieved a more competitive performance against other remote sensing dehazing methods on commonly used benchmark datasets.

## 1. Introduction

In recent years, the rapid development of remote sensing image technology has greatly improved the quality of remote sensing images, which can be applied in many fields, such as building extraction, urban planning, disaster management, and military reconnaissance. The performances of these applications mainly depend on clean remote sensing data. Considering that remote sensing images are captured from aerial platforms, they are susceptible to low visibility, color shift, and blurring due to varying densities of haze, resulting in a significant decrease in data availability. Hence, there is an urgent need to develop a method that can effectively remove haze disturbances in remote sensing images. Working toward this goal, traditional methods explore various interpretable hand-crafted priors in different solution spaces. The widely used priors can be summarized as virtual point clouds [1], dark channel priors [2], frequency dependence, etc. However, these hand-crafted priors only take effect in some limited scenarios and are not universally applicable, often leading to poor restoration performances [3]. The emergence of deep learning has further promoted research on remote sensing image restoration. By exploiting the powerful feature-modeling ability of convolutional neural networks (CNNs), many deep learning-based remote sensing image-dehazing methods [4,5,6,7,8,9,10,11] have been developed, such as conditional generative adversarial networks, unsupervised learning [12], and channel refinement. These approaches formulate the dehazing task as the process of pixel regression, directly learning the image reconstruction from hazy to clear through the model. Due to severe degradation, degraded images often contain severe blurring, and frequency domain information includes structure and edges, which is important for restoring clear images. However, most existing methods [4,6,13,14,15,16] explore spatial information to recover edges from degraded images, while frequency domain information is often overlooked in these methods.

**Figure 1:**
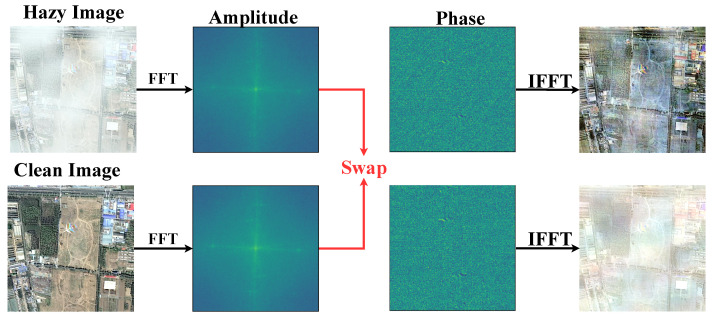
We swapped the amplitude of the hazy image with the amplitude of the paired clean image, and it can be seen that the original hazy image became clear, while the clear image became blurred.

Encouraged by previous Fourier-based work [17,18,19], we explored the properties of Fourier frequency information for remote sensing dehazing tasks, as reported in Figure 1. As shown in Figure 1, given two images (remote sensing hazy images and their paired clear images), we swapped their amplitude components and combined them with the corresponding phase components in Fourier space. The reconstruction results indicate that after the amplitude swap, the visual appearance of the degraded images underwent an exchange, evidencing that the degradation information of degraded images was mainly included in the amplitude component. Therefore, how to fully utilize the properties of frequency domain information and effectively integrate it into remote sensing image dehazing models it is a prominent problem.

Recently, due to the ability to capture the long-distance dependencies of features, Transformer-based methods [20,21,22] have gradually been applied to remote sensing image-dehazing tasks. These methods rely on the hierarchical encoding and decoding structure and self-attention to learn the mapping of potentially clear images in the network. In order to address the high computational burden brought about by self-attention in Transformers, recent methods have adopted compressing the computational range of attention or utilizing approximate matrix calculations to reduce the computational complexity of traditional self-attention. Although these methods were demonstrated to be effective in improving computational efficiency, they cannot effectively capture the long-range dependencies of features due to sacrificing the representation ability of the network, resulting in significant artifacts and loss of image texture, especially when processing high-resolution images. The standard self-attention [23,24] captures long-range dependencies by calculating the similarity between one token and other tokens. In fact, according to the convolution theorem [25,26], this calculation process can be achieved by transforming tokens into the frequency domain and utilizing the Hadamard product of elements when rearranging them. Combining Figure 1 and the above findings naturally raises a question: *Can we coordinate the characteristics of degraded information in the frequency domain with the above findings to achieve high-quality remote sensing image dehazing*?

In this paper, we propose a frequency-oriented transformer for remote sensing image dehazing called FOTformer. This network consists of three key designs: a frequency-prompt attention evaluator (FPAE), content reconstruction feed-forward network (CRFN), and spatial-frequency aggregation block (SFAB). FPAE first computes the self-correlation of the feature in the frequency domain to replace traditional spatial self-attention and to learn complex details and comprehensive features in the model. Note that FPAE calculates the correlation between an element in the query and all elements in the key through a Hadamard product in the frequency domain rather than matrix multiplication in the spatial domain. Subsequently, to enhance the representation ability, we introduce prompt components [14,27] in FPAE to adaptively guide the model to focus on more important information. CRFN extracts information from different scales in features, integrates and processes global frequency domain information and local multi-scale spatial information in Fourier space, and reconstructs global content under the guidance of the amplitude spectrum. In addition, we propose the SFAB to interact and aggregate information between the frequency domain and spatial domain. This block can facilitate the broadcast of features from the encoder to the decoder, alleviating the problem of information loss.

In summary, the main contributions of our work are as follows:

- We developed a frequency-prompt attention evaluator to learn complex details and comprehensive features in the model in the frequency domain. This evaluator can effectively aggregate relevant features in the frequency domain rather than the spatial domain, thereby improving the image restoration performance.
- We propose a content reconstruction feed-forward network that captures information between different scales in features and integrates and processes global frequency domain information and local multi-scale spatial information in Fourier space to reconstruct global content under the guidance of the amplitude spectrum.
- We designed a spatial-frequency aggregation block to exchange and fuse features from the frequency domain and spatial domain of the encoder and decoder, promoting the propagation of features from the encoder stream to the decoder and alleviating the problem of information loss in the network.

## 2. Related Work

In this section, we introduce the related works on image dehazing and visual prompt learning.

### 2.1. Image Dehazing

Previous research in the field of image dehazing mainly focused on ground landscapes, which can be divided into methods using prior-based atmospheric scattering models (ASM) [2,5,28] and deep learning-based methods using deep learning. To combat the image distortion caused by prior estimation errors, end-to-end deep learning-based dehazing methods have become the mainstream approach, directly transitioning from foggy to clear images. This type of method increases the flexibility and adaptability to complex haze conditions. Specifically, Li et al. [29] proposed AOD-Net by reconstructing the atmospheric scattering model directly and achieved superior image clarity. Mei et al. [30] proposed an input-adaptive trainable end-to-end dehazing model (PFFNet), circumventing the complexities of realistic atmospheric conditions and providing direct learning of a highly nonlinear transformation from observed hazy images to the haze-free ground truth. Qin et al. [31] presented a feature fusion attention network (FFA-Net), which can restore image details and color fidelity by retaining shallow information. Dong et al. incorporated dense skip connections based on the U-Net architecture for better information flow. Hayat Ullah et al. [32] proposed a computationally efficient lightweight convolutional neural network called Light-DehazeNet (LD-Net) for reconstructing hazy images in surveillance and industrial sectors affected by weather conditions by jointly estimating the transmission map and atmospheric light to improve image quality and productivity. Recently, Transformer models have made breakthroughs in computer vision and many modified Transformer architectures have been proposed for low-level vision tasks. For example, Song et al. [13] made some improvements to the normalization layer and activation function based on the Swin Transformer to adapt image dehazing. Wang et al. [33] employed the Uformer architecture, which features a locally enhanced window (LeWin) Transformer block and a learnable multi-scale restoration modulator, for effective and efficient image restoration, capturing both local and global dependencies.

In recent years, some dehazing methods tailored for satellite images have been explored. Li et al. [34] presented a two-stage dehazing network (FCTF-Net) for haze removal tasks on satellite images by performing coarse dehazing and then refining the results for an enhanced performance. Guo et al. [7] proposed SCANet, which is a self-paced semi-curricular attention network, for effectively handling non-homogeneous haze in image dehazing by focusing on enhancing haze-occluded regions. Ashutosh Kulkarni et al. [22] presented a novel approach (AIDNet) for satellite image dehazing, utilizing deformable multi-head attention with spatially attentive offset extraction and edge-boosting skip connections to preserve minute textures and enhance the accuracy in visual data-dependent applications. Song et al. [21] presented an effective remote sensing image-dehazing Transformer architecture named RSDformer to capture both local and non-local features. It is worth noting that most existing transformer methods rely on self-attention based on spatial matrix multiplication for feature aggregation, which leads to significant drawbacks, such as high computational costs and difficulty in deploying models. In contrast to the above works, we adopted the element-wise Hadamard product in the frequency domain to evaluate the autocorrelation of features in order to reduce the computational overhead caused by original spatial self-attention.

### 2.2. Visual Prompt Learning

The emergence of prompt learning in natural language processing [35,36,37] has led to rapid progress in its application to vision-related tasks, with recent studies also considering finding the right prompt for low-level vision models [27,38,39]. The goal of this work was not to explicitly prompt the model with the specific degradation type and degree to address the remote sensing image dehazing problem.

In contrast to previous methods, our method does not directly utilize pretrained models to generate raw degraded features. Instead, we delved into degradation-specific information to achieve better image restoration results. We propose prompting the restoration model from a frequency domain perspective. By leveraging frequency domain features as prompts, our model integrates and processes global frequency domain information, reconstructing global content under the guidance of the magnitude spectrum. This tailored extraction ensures that the model focuses on specific image features that are directly relevant to remote sensing image dehazing.

## 3. Proposed Methods

In this section, we first provide an overview of the proposed FOTformer, and then offer a detailed report of the developed frequency-prompt attention evaluator, content reconstruction feed-forward network, and spatial-frequency aggregation block.

### 3.1. Overview

**Figure 2:**
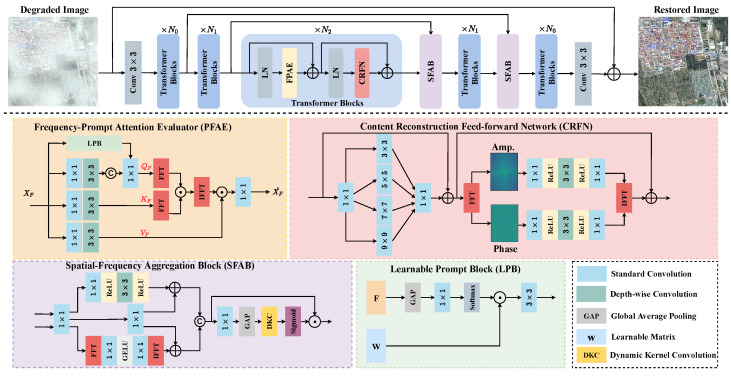
The architecture of the proposed frequency-oriented transformer for remote sensing image dehazing, which takes remote sensing hazy images as the input and generates dehazed images as the output. It mainly contains the frequency-prompt attention evaluator (FPAE), content reconstruction feed-forward network (CRFN), and spatial-frequency aggregation block (SFAB).

The overall pipeline of our proposed FOTformer, as shown in Figure 2, is based on a three-level encoder–decoder framework, with the aim of solving the issue of remote sensing image dehazing. To achieve multi-scale representation, each layer of the encoder–decoder is configured with a specific spatial resolution and channel size. First, a 3 × 3 convolution is used to obtain shallow feature embeddings to extract essential information from the image. Subsequently, frequency modulation Transformer blocks are stacked throughout the entire architecture pipeline to learn feature maps for removing haze and reconstructing clear images. Finally, 3 × 3 convolution is introduced to project the learned features onto the original size. In the entire pipeline, the critical blocks of FOTformer include the frequency-prompt attention evaluator (FPAE), content reconstruction feed-forward network (CRFN), and spatial-frequency aggregation block (SFAB). These blocks are responsible for processing self-correlation relationships between features, global frequency information, and local multi-scale spatial information. By interacting and aggregating information between the frequency and spatial domains, high-quality and clear image reconstruction is achieved. We exploited pixel shuffling and pixel shuffle operations to achieve downsampling and upsampling of features in the pipeline. The key designs mentioned above are elaborated in detail in the next section.

### 3.2. Frequency-Prompt Attention Evaluator

We first concisely describe the calculation process of standard self-attention in traditional Transformers, and then provide a detailed report on the proposed frequency-prompt attention evaluator (PFAE). Consider an input $X\in R^{H\times W\times C}$, where the H and W indicate the height and width of the input, and c represents the channel size. We first reshape it to $X\in R^{N\times C}$, where N=H×W. Then, linear projection matrices $W^{Q}$, $W^{K}$, and $W^{V}$ are used to generate the query (Q), key (K), and value (V). The calculation process can be expressed as follows:

(1) $$\begin{array}{cc} Q,K,V & =W^{Q}X,W^{K}X,W^{V}X, \end{array}$$

According to the gained Q, K, and V, traditional self-attention can be defined as follows:

(2) $$\begin{array}{cc} {\mathrm{Att}}_{X} & =\mathrm{softmax}\left(\frac{Q\cdot K^{T}}{\alpha }\right)\cdot V, \end{array}$$

where the ${\mathrm{Att}}_{X}$ denotes the attention map, and α indicates a learnable parameter for controlling the size of the dot product value.

Due to the involvement of spatial matrix multiplication in this calculation process, it is obvious that the spatial complexity of the model is intolerable. The consumption of computing resources is unacceptable when processing high-resolution images. In addition, the model only focuses on the self-correlation of features in space during the calculation process, ignoring the potential frequency information representation in the model, which may limit the restoration of image details and texture structures.

We note that the standard self-attention captures long-range dependencies by calculating the similarity between one token and other tokens. Inspired by the convolution theorem and previous work, this calculation process can actually be achieved by transforming the token to the frequency domain and exploiting the Hadamard product between the tokens when rearranging these tokens.

Working toward this, we propose an effective frequency-prompt attention estimator. Specifically, the element-wise Hadamard product is applied in the frequency domain to evaluate the similarity and potential frequency representation between elements, rather than matrix multiplication in space. Similar to standard self-attention, given an input $X_{F}$, a 1×1 convolution and 3×3 depth-wise convolution are used to find the query ($Q_{F}$), key ($K_{F}$), and value ($V_{F}$). This calculation process can be defined as follows:

(3) $$\begin{array}{c} Q_{F}^{\prime },K_{F},V_{F}={\mathrm{DWConv}}_{3\times 3}\left({\mathrm{Conv}}_{1\times 1}\left(X_{F}\right)\right). \end{array}$$

Prompt-based techniques have been widely used to enhance network contextual information. Therefore, in FPAE, learnable prompt blocks (LPBs) are used to enrich the degradation information captured in the network by interacting with the input feature F. Specifically, an LPB first adopts the global average pooling operation on the input feature F to generate a global representation $G_{i}$ across spatial dimensions. Then, a 1×1 convolution is applied to compress $G_{i}$ to obtain compact feature vectors. Next, softmax is executed to obtain weights. Finally, the interaction between this weight and the learnable matrix w is conducted to enrich the contextual information. This procedure can be formulated as follows:

(4) $$\begin{array}{cc} G_{i} & =\mathrm{Softmax}\left({\mathrm{Conv}}_{1\times 1}\left(\mathrm{GAP}\left(F\right)\right)\right),\\ G_{F} & ={\mathrm{Conv}}_{3\times 3}\left(W⊙G_{i}\right), \end{array}$$

where $G_{F}$ denotes the final output. After obtaining $G_{F}$, it is combined with $Q_{F}^{\prime }$ to form $Q_{F}$:

(5) $$\begin{array}{cc} Q_{F} & ={\mathrm{Conv}}_{1\times 1}\left(\mathrm{concat}\left(Q_{F}^{\prime },G_{F}\right)\right), \end{array}$$

where term “concat” presents feature concat operation.

A fast Fourier transform (FFT) operation is performed on $Q_{F}$ and $Q_{K}$ to evaluate their correlation in the frequency domain. Note that fast Fourier transforms are only performed on $Q_{F}$ and $Q_{K}$. First, the distribution of haze is sweeping and non-uniform, rather than limited to a specific frequency range in remote sensing image-dehazing tasks. If the FFT is also applied to $Q_{V}$, it may implicitly exacerbate the degradation of image quality and reduce the effectiveness of the image restoration. This procedure can be formulated as follows:

(6) $$\begin{array}{c} {\mathrm{Att}}_{F}=F^{-1}\left(F\left(Q_{F}\right)⊙{\left(F\left(K_{F}\right)\right)}^{H}\right), \end{array}$$

where F and $F^{-1}$ denote the FFT and inverse FFT operations, respectively. ${\left(\cdot \right)}^{H}$ presents the conjugate transpose operation and ⊙ indicates the Hadamard product. Finally, features are aggregated by interacting with $V_{F}$:

(7) $$\begin{array}{cc} \hat{X_{F}} & ={\mathrm{Conv}}_{1\times 1}\left({\mathrm{Att}}_{F}⊙V_{F}\right), \end{array}$$

where ${\mathrm{Conv}}_{1\times 1}$ is applied to fuse the obtained feature.

### 3.3. Content Reconstruction Feed-Forward Network

Previous works typically applied single-scale depth-wise convolution in the feed-forward network to enhance the ability of the model to learn local features. Nevertheless, this design is subject to the limited receptive field and makes it difficult to achieve high-quality content reconstruction. In fact, local and global representations are vital for obtaining high-quality content reconstruction. Therefore, multi-scale encoding is first performed within the input features and dynamically model the representation of multi-scale spatial information. Then, we utilized the fact that the Fourier transform can handle global information, where the captured multi-scale feature representations are transformed into the frequency domain for implementing global information encoding. Specifically, in the spatial domain, a 1×1 convolution is used to perform deep feature mapping on the input feature $F_{c}$ in proportion to *s*. Then, it is divided into four parts along the channel direction and multi-scale convolution is exploited to explicitly expand the receptive field within the features, obtaining the spatial feature $S_{l}$. Subsequently, a 1×1 convolution is employed to restore $S_{l}$ to its original input size. After obtaining the rich space representation, FFT is used to transform it into the frequency domain. As reported in Figure 1, the degradation factors in the degraded image are concentrated in the amplitude component, while structural and detailed information is included in the phase component. Therefore, two layers of 1×1 convolution and one layer of 3×3 depth-wise convolution in different components are used to separately learn potential clear feature maps. Next, IFFT is performed to map it to the spatial domain and obtain the frequency domain feature $F_{g}$. To stabilize the model training, residual learning is applied in both the spatial feature modeling and frequency domain feature modeling processes. Overall, the procedure can be formulated as follows:

(8) $$\begin{array}{cc} \; & F_{1},F_{2},F_{3},F_{4}=\mathrm{split}\left({\mathrm{Conv}}_{1\times 1}\left(F_{c}\right)\right),\\ \; & S_{l}={\mathrm{Conv}}_{1\times 1}\left({\mathrm{Conv}}_{k\times k}\left(F_{1},F_{2},F_{3},F_{4}\right)\right)+F_{c},\\ \; & F_{AMP},F_{AMP}=\mathrm{split}\left(F\left(S_{l}\right)\right),\\ \; & \bar{F_{AMP}}={\mathrm{Conv}}_{1\times 1}\left(\sigma \left({\mathrm{DConv}}_{3\times 3}\left(\sigma \left({\mathrm{Conv}}_{1\times 1}\left(F_{AMP}\right)\right)\right)\right)\right),\\ \; & \bar{F_{PHA}}={\mathrm{Conv}}_{1\times 1}\left(\sigma \left({\mathrm{DConv}}_{3\times 3}\left(\sigma \left({\mathrm{Conv}}_{1\times 1}\left(F_{PHA}\right)\right)\right)\right)\right),\\ \; & F_{g}=F^{-1}\left(\mathrm{concat}\left(\bar{F_{AMP}},\bar{F_{PHA}}\right)\right)+S_{l}, \end{array}$$

where σ denotes the ReLU nonlinear activation function.

### 3.4. Spatial-Frequency Aggregation Block

We observed that most existing methods typically exploit skip connections or element concat operations to merge features of different scales in the network. Due to being affected by degradation and redundant feature information, this may lead to sub-optimal removal results [40]. To this end, we propose a spatial-frequency aggregation block (SFAB) that extracts features from both the spatial and frequency domains, facilitating feature propagation between the encoder and decoder and alleviating information loss. In this model, we adopted it to replace traditional skip connections to generate enhanced features $F_{O}$ to promote high-quality image restoration. The calculated procedure of the SFAB can be equated mathematically as follows:

(9) $$\begin{array}{cc} F_{SP} & ={\mathrm{Conv}}_{1\times 1}(\mathrm{concat}\left(F_{en},F_{de}\right)),\\ F_{S1} & =\sigma ({\mathrm{DConv}}_{3\times 3}\left(\sigma \left({\mathrm{Conv}}_{1\times 1}\left(F_{SP}\right)\right)\right)),\\ F_{S2} & =F^{-1}\left({\mathrm{Conv}}_{1\times 1}\left(\tau \left({\mathrm{Conv}}_{1\times 1}\left(F\left(F_{SP}\right)\right)\right)\right)\right),\\ F_{S3} & ={\mathrm{Conv}}_{1\times 1}(F_{S1}),\\ F_{M} & =\mathrm{concat}(F_{S3}+F_{S1},F_{S3}+F_{S2}),\\ F_{O} & =\varphi \left(\mathrm{DKC}(\mathrm{GAP}({\mathrm{Conv}}_{1\times 1}\left(F_{M}\right)))\right)⊙F_{M} \end{array}$$

where φ and τ denote the sigmoid and GELU nonlinear activation functions, respectively.

In traditional CNNs, it is worth noting that the size of the convolution kernel used to capture features is fixed. This means that some features may be overly smoothed (when the kernel size is large) or insufficiently smoothed (when the kernel size is small) given a fixed kernel size, resulting in the loss of important information and reducing the performance. To address this issue, we adopted dynamic kernel convolution (DKC) [41], which dynamically selects the size of the convolution kernel based on the number of input feature channels. Specifically, the size of the convolution kernel is dynamically determined by applying a learnable 1D convolution layer to the input features, and then this layer is used to measure the features of each channel. Through this strategy, the network can adaptively select kernels of different sizes to capture the features of each channel in the input. The dynamic kernel size can be determined by the following formula:

(10) $$\begin{array}{c} k=\alpha \left(C^{\prime }\right)={\left|\frac{\log_{2}\left(C^{\prime }\right)}{n}+\frac{m}{n}\right|}_{\mathrm{odd}}, \end{array}$$

where $C^{\prime }$ represents the number of channels for the feature after performing the global average pooling operation, and |f| denotes the odd number closest to *f*. This study set *m* and *n* to 1 and 2, respectively.

### 3.5. Loss Functions

To better recover more similar structure details, we applied the $L_{1}$ loss as the reconstruction loss and trained the model by minimizing it. The loss function can be depicted as follows:

(11) $$\begin{array}{c} L_{1}\left(I_{\mathrm{dehaze}},I_{\mathrm{gt}}\right)\;={∥I_{\mathrm{gt}}\;-I_{\mathrm{dehaze}}∥}_{1}. \end{array}$$

Due to the difference in frequency domain distribution between haze images and clean images, we also imposed restrictions in the frequency domain. Specifically, we first performed the FFT operation on the dehazing image and ground truth image, then estimated the average absolute error between them. This calculation process can be defined as follows:

(12) $$\begin{array}{c} L_{\mathrm{fft}}\left(I_{\mathrm{dehaze}\;},I_{\mathrm{gt}}\right)\;={∥F\left(I_{\mathrm{gt}}\right)-F\left(I_{\mathrm{dehaze}}\right)∥}_{1}. \end{array}$$

To effectively preserve the structural details in the image, we also introduced edge loss. It can be defined as follows:

(13) $$\begin{array}{c} L_{\mathrm{edge}}\left(I_{\mathrm{dehaze}},\;I_{\mathrm{gt}}\right)\;=\sqrt{{∥I_{\mathrm{gt}}\;-I_{\mathrm{dehaze}}∥}_{2}+\varepsilon^{2}}. \end{array}$$

Overall, the whole loss function is defined as follows:

(14) $$\begin{array}{c} L_{\mathrm{total}}=\lambda_{1}L_{1}+\lambda_{2}L_{\mathrm{fft}}+\lambda_{3}L_{\mathrm{edge}}, \end{array}$$

where the $\lambda_{1}$, $\lambda_{2}$, and $\lambda_{3}$ are all set to 1.

## 4. Experimental Results

To demonstrate the effectiveness of the proposed FOTformer, we first evaluated the performance of our method on different benchmark datasets, including the SateHaze1k, RICE, and RRSD300 datasets. Then, we also conducted ablation experiments to evidence the contribution of each component.

### 4.1. Datasets

To fully estimate the performance of the proposed method, synthetic and real-world datasets were used, which contained the following:

(1) SateHaze1k: SateHaze1k [1] is the commonly employed synthetic dataset consisting of three subsets: thin haze, moderate haze, and thick haze. Each subset contains 400 pairs of synthetic remote sensing RGB haze images, where the resolution of all images is 512×512; 320 pairs were adopted for training and 80 pairs were used for testing.
(2) RICE: RICE [34] was proposed by Google Earth for remote sensing image cloud removal tasks. This dataset consists of 500 paired remote sensing RGB hazy images, of which 425 pairs were used for training and 75 pairs were used for testing. The same as SateHaze1k, the resolution of the images in RICE is 512×512.
(3) RRSD300 [42]: To further demonstrate the universality of the proposed method, we conducted experiments on the real-world dataset RRSD300. RRSD300 is a dataset containing 300 real remote sensing haze images and not containing paired clear images. These images were captured from remote sensing platforms in the real world, which includes dense and non-uniform haze scenes.

### 4.2. Compared Methods

We compared FOTformer with a prior-based method (DCP [2]), CNN-based dehazing baselines (DehazeNet [4], AODNet [29], PFFNet [30], FFA-Net [31], FCTF [34], MSBDN [43], LD-Net [32], SCANet [7]), and recent Transformer-based methods (Dehazeformer [13], UFormer [33], AIDNet [22], and RSDformer [21]). For recent dehazing baselines, for instance, UFormer, AIDNet, and RSDformer, if no pre-training weights are provided, we retrained the baseline provided by the author. Otherwise, we evaluated these methods for fair comparison through the online code provided by the author.

### 4.3. Implementation Details

During the training process, we conducted the proposed network in the PyTorch framework with an Adam optimizer and a batch size of 4. We uniformly cut the data into 128 patch sizes for training and applied the sliding-window-slicing strategy for testing. We set the learning rate to $2\times 10^{-4}$ and applied the cosine annealing strategy to steadily decrease the final learning rate to $1\times 10^{-6}$. For the SateHaze1k and RICE datasets, we trained the model with 1000 epochs. We set the stacking numbers of FMTB $\left(N_{0},N_{1},N_{2}\right)$ to [4,8,8] in the model. All experiments were executed on an NVIDIA GeForce RTX 3080Ti GPU (12GB).

### 4.4. Evaluation Metrics

Following the previous work, we calculated the values of the peak signal-to-noise ratio (PSNR) [44] and structural similarity index (SSIM) [45] for the predictions and ground truth as evaluation metrics in the RGB channel. The PSNR can be defined as follows:

(15) $$\begin{array}{c} \mathrm{PSNR}=10\cdot \log_{10}\left(\frac{{\mathrm{MAX}}^{2}}{\mathrm{MSE}}\right), \end{array}$$

where MAX is the maximum value of the image pixels, and the mean squared error (MSE) was adopted to calculate the mean square difference between the predicted clear image and its paired clear image. The SSIM can be defined as follows:

(16) $$\begin{array}{c} \mathrm{SSIM}\left(x,y\right)=\frac{\left(2\mu_{x}\mu_{y}+C_{1}\right)\left(2\sigma_{xy}+C_{2}\right)}{\left(\mu_{x}^{2}+\mu_{y}^{2}+C_{1}\right)\left(\sigma_{x}^{2}+\sigma_{y}^{2}+C_{2}\right)}, \end{array}$$

where *x* and *y* denote the predicted clear image and its paired clear image, respectively. $u_{x}$ and $\sigma_{x}^{2}$ denote the mean and variance of *x*, respectively, while $\sigma_{xy}$ denotes the covariance of *x* and *y*. $C_{1}$ and $C_{2}$ are constants to maintain the stability of the equation. Usually, SSIM takes a value from 0 to 1. In the image deraining problem, a higher value of SSIM obtained represents a more effective result of image restoration.

In addition, learned perceptual image patch similarity (LPIPS) [46] was adopted to evaluate the similarity between the predicted images and the ground truth. The LPIPS can be defined as follows:

(17) $$\begin{array}{c} \mathrm{LPIPS}\left(x,y\right)=\frac{1}{N}\sum_{i=1}^{N}{\left|\phi \left(x_{i}\right)-\phi \left(y_{i}\right)\right|}_{2}, \end{array}$$

where $x_{i}$ and $y_{i}$ represent the *i*-th perceptual feature of *x* and *y*, respectively. ϕ(·) is a neural network used to extract perceptual features. ${\left|\cdot \right|}_{2}$ represents the $L_{2}$ norm. *N* denotes the number of perceptual features.

### 4.5. Main Results

**Figure 3:**
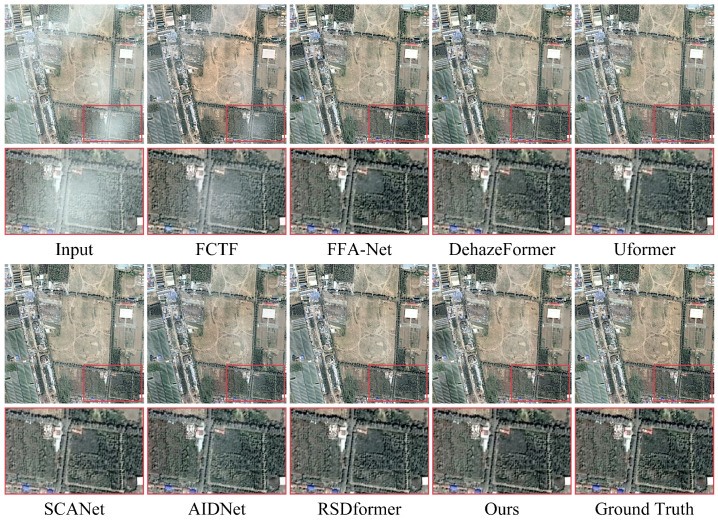
Visual comparison of the results from the thin haze SateHaze1k dataset. They are best viewed by zooming in on the figures on high-resolution displays.

**Figure 4:**
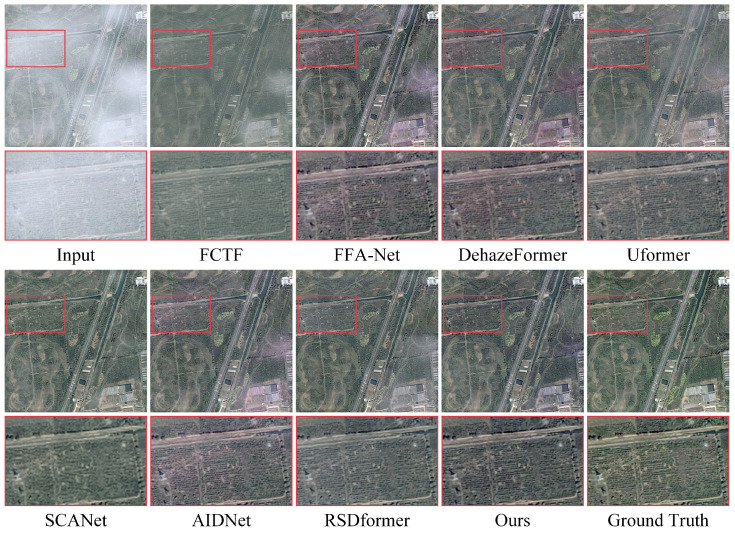
Visual comparison of the results from the moderate haze SateHaze1k dataset. They are best viewed by zooming in on the figures on high-resolution displays.

**Figure 5:**
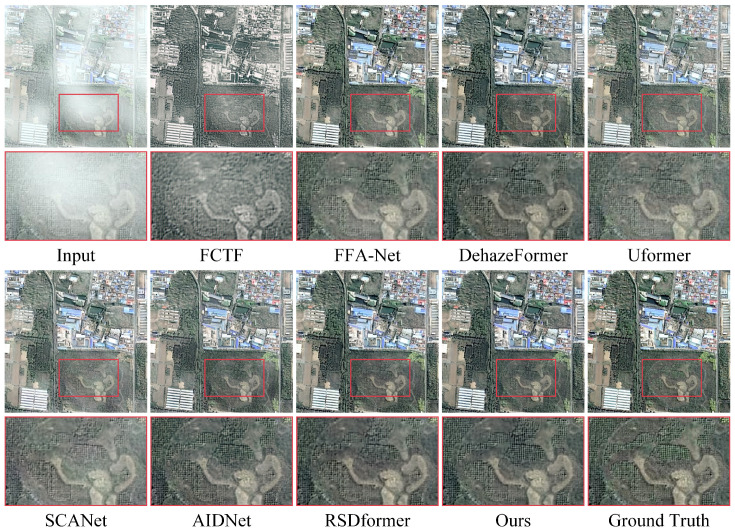
Visual comparison of the results from the thick haze SateHaze1k dataset. They are best viewed by zooming in on the figures on high-resolution displays.

**Synthetic datasets:** Table 1 and Table 2 provide a comprehensive comparison between our proposed method and 13 representative and competitive dehazing methods. It is evident that capturing the potential frequency information could significantly improve the performance in terms of PSNR and SSIM values compared with all the other baselines. Notably, our approach achieved more appealing results on the thin haze benchmark of SateHaze1k, surpassing the recent CNN-based method SCANet by 3.57 dB in the PSNR. Compared with the recent Transformer-based methods AIDNet and RSDformer, the proposed method attained 1.53 dB and 1.11 dB gains in terms of the PSNR, respectively. The performance improvement, when compared with existing remote sensing dehazing methods, shows that learning the frequency information from the frequency domain can facilitate high-quality image dehazing results. In addition, Figure 3, Figure 4 and Figure 5 show the qualitative comparison with other dehazing methods on the SateHaze1k dataset. As expected, it can be observed that SCANet, UFormer, and AIDNet failed to fully remove dense haze, showing contrast decline and color distortion. However, recent typical image restoration methods, such as RSDformer and FFA-Net, could obtain higher-quality images. Unfortunately, these still produced residual artifacts after haze removal and could not fully restore the color and edge details. Compared with these competitive methods, the proposed FOTformer preserved more details and achieved excellent perceptual quality.

**Table 1:** Comparison of quantitative results on the SateHaze1k dataset. **Bold** and underline denote the best and second-best results.

Benchmark Datasets		Thin Haze [22]			Moderate Haze [22]			Thick Haze [22]		
Metrics		PSNR (dB)↑	SSIM↑	LPIPS↓	PSNR (dB)↑	SSIM↑	LPIPS↓	PSNR (dB)↑	SSIM↑	LPIPS↓
Prior-based method	DCP [2]	13.259	0.7445	0.2423	9.64	0.6141	0.3938	10.45	0.6110	0.3786
CNN-based methods	DehazeNet [9]	16.57	0.4887	0.7493	16.93	0.2992	0.8673	15.44	0.3689	0.8138
	AODNet [29]	18.74	0.8584	0.0991	17.69	0.7969	0.3143	13.41	0.6523	0.4746
	PFFNet [30]	18.02	0.6689	0.5881	18.06	0.5487	0.5265	15.06	0.3369	0.7877
	FFA-Net [31]	24.26	0.9102	0.0681	25.39	0.9302	0.0852	21.83	0.8361	0.1616
	FCTF [34]	19.54	0.8528	0.1348	18.41	0.7314	0.2875	17.11	0.7205	0.5835
	MSBDN [43]	21.76	0.8812	0.0873	23.59	0.8877	0.1034	20.21	0.7959	0.2254
	LD-Net [32]	20.24	0.8739	0.0844	19.40	0.7370	0.2616	18.62	0.7803	0.1862
	SCANet [7]	21.75	0.8587	0.1210	21.39	0.7290	0.4166	19.32	0.8007	0.1914
Transformer-based methods	DehazeFormer [13]	23.25	0.8996	0.0654	25.38	0.9282	0.0738	22.60	0.8366	0.1579
	UFormer [33]	21.68	0.8885	0.0745	21.14	0.8321	0.1399	19.88	0.8062	0.1901
	AIDNet [22]	23.79	0.8942	**0.0603**	25.15	0.9032	**0.0414**	20.60	0.8149	**0.1281**
	RSDformer [21]	24.21	0.9118	0.0677	26.24	0.9341	0.0657	**23.01**	**0.8528**	0.1576
	Ours	**25.34**	**0.9170**	0.0517	**26.32**	**0.9419**	0.0608	**23.24**	0.8503	**0.1157**

**Figure 6:**
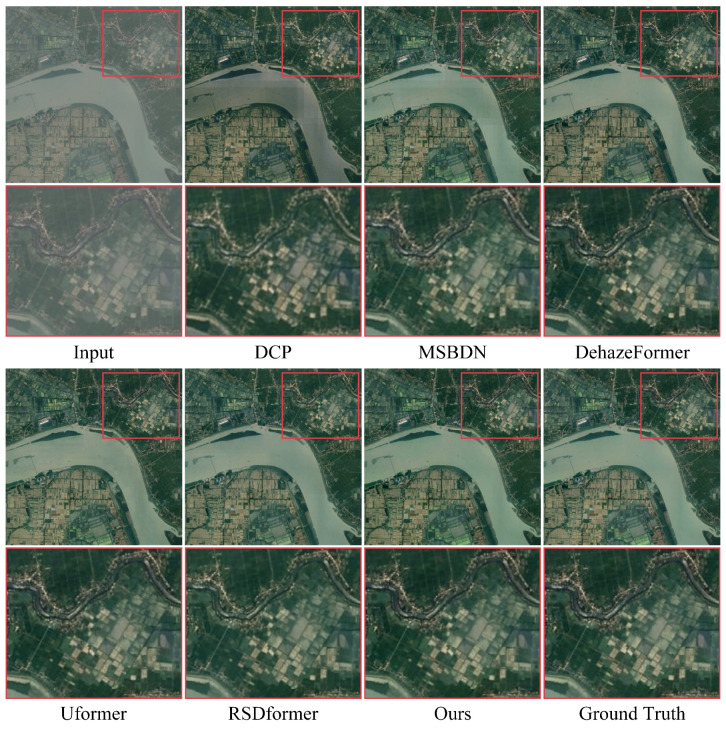
Visual comparison of the results from the RICE dataset. They are best viewed by zooming in on the figures on high-resolution displays.

We also exhibit the visual comparison with other dehazing methods on the RICE dataset in Figure 6. The visual comparison indicates that our method exhibited enhanced contrast and reduced color distortion in comparison with the other approaches.

**Table 2:** Comparison of quantitative results on the RICE dataset. **Bold** and underline denote the best and second-best results.

Benchmark Datasets		RICE Dataset [34]		
Metrics		PSNR (dB)↑	SSIM↑	LPIPS↓
Prior-based method	DCP [2]	17.48	0.7841	0.1794
CNN-based methods	DehazeNet [9]	-	-	-
	AODNet [29]	23.77	0.8731	0.1469
	PFFNet [30]	25.64	0.8977	0.1975
	FFA-Net [31]	28.54	0.9396	0.0755
	FCTF [34]	16.57	0.8847	0.1567
	MSBDN [43]	30.37	0.8584	0.0991
	LD-Net [32]	28.88	0.9336	0.0897
	SCANet [7]	30.84	0.9433	0.0689
Transformer-based methods	DehazeFormer [13]	30.91	0.9350	0.0721
	UFormer [33]	32.13	0.9413	**0.0590**
	AIDNet [22]	-	-	-
	RSDformer [21]	33.01	0.9525	0.0675
	Ours	**33.39**	**0.9537**	0.0606

**Figure 7:**
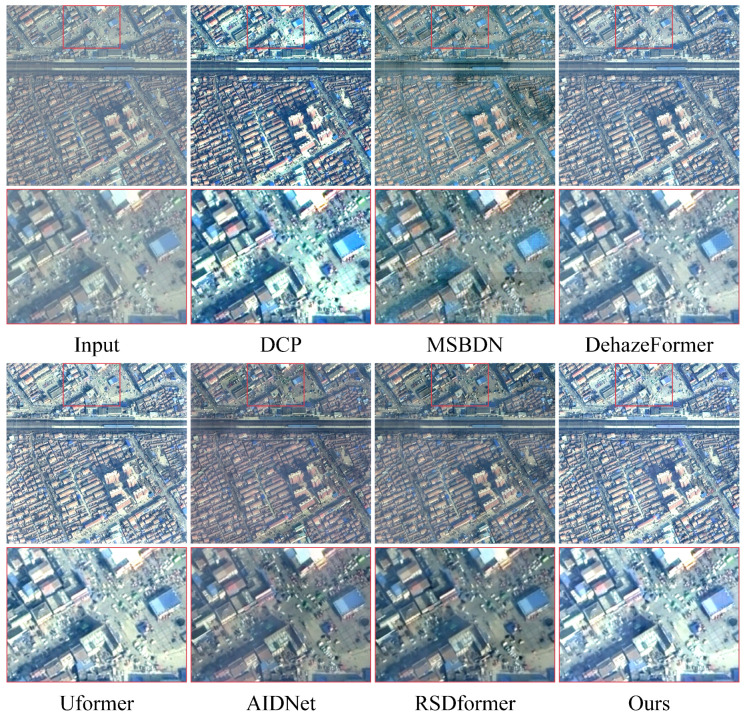
Visual comparison of the results from the RRSD300 dataset. They are best viewed by zooming in on the figures on high-resolution displays.

**Real-world datasets:** To further evaluate the qualitative performance, we conducted additional experiments using the RRSD300 benchmark dataset. The visual results are reported in Figure 7. The results demonstrate that most models had difficulty in effectively dealing with large-range and non-uniform distributions of real-world haze, resulting in noticeable haze effects in their outputs. In contrast, our model achieved impressive remote sensing dehazing results compared with the other comparative models. The proposed model could effectively eliminate a significant portion of the haze disturbances, resulting in visually pleasing restoration effects. This indicates that in real remote sensing dehazing scenarios, our network exhibited superior output quality with clearer content and enhanced perceptual quality.

### 4.6. Model Efficiency

Table 3 delineates a comparative analysis between FOTformer and the other foundational models, encompassing both CNN-based and Transformer-based methods, concerning parameters and FLOPs. Notably, FOTformer distinguished itself by its comparatively diminutive computational overhead. In contrast to Transformer-based architectures, like Uformer, FOTformer evinced marginally reduced FLOPs while concurrently obtaining superior PSNR and SSIM values. Conversely, in comparison with CNN-based approaches, FOTformer manifested superior dehazing outcomes with relatively constrained parameters. Overall, the proposed FOTformer exhibited a competitive performance compared with existing remote sensing dehazing baselines.

### 4.7. Ablation Studies

We conducted the ablation experiments to better understand the impacts of the FPAE, CRFN, SFAB, and loss functions on the performance. Unless otherwise specified, all ablation experiments were conducted on the thin haze SateHaze1k dataset using the experimental setup described in Section 4.3.

**Effectiveness of different components**: In this section, we discuss the effectiveness of components in FOTformer. Table 4 shows the quantitative results of the model on the thin haze dataset. The different models are described as follows: (Baseline) The same framework and settings as FOTformer were used, where we applied MDTA [47] and FFN [13] as the basic components. (a) MDTA was replaced with the proposed FPAE. (b) MDTA was kept and FFN was replaced with CRFN. (c) MDTA and FFN were repalced with FPAE and CRFN, respectively. (d) MDTA was replaced with FPAE and SFAB was added. (e) FFN was replaced with CRFN and SFAB was added. (f) The proposed FOTformer. It can be observed that for the baseline, FPAE and CRFN provided PSNR performance gains of 1.62 dB and 1.14 dB, respectively. This indicates that FPAE and CRFN were indispensable components in our model that were capable of effectively learning degradation information related to haze in images, thereby eliminating haze disturbances and reconstructing clear images. Relative to model (b), model (e) achieved a performance improvement of 0.39 dB by adding SFAB. This indicates that combining frequency information and spatial information could effectively enhance the feature modeling ability of the model. The performance of model (f), i.e., the proposed FOTformer, was the best, obtaining the better PSNR and SSIM and lower LIIPS values. This indicates that the collaboration of the three designs could achieve the best remote sensing image dehazing effect.
**Effectiveness of different loss functions**: To further reduce the difference between the dehazing images and ground-truth images, we explored the impacts of different loss functions on the proposed network. Table 5 reports the experimental results. It can be significantly observed that the combined performance of various loss functions is better than a single loss function.
**Effectiveness of designs of the FPAE and CRFN**: To enrich the contextual features, we introduced learnable prompt blocks (LPBs). We conducted experiments on FOTformer using and not using LPBs to verify the effectiveness of the design. The experimental results are shown in Table 6, and it can be observed that the FOTformer using LPBs achieved higher PSNR values, which demonstrated that LPBs could allow the model to remove haze disturbances in the image. In addition, to verify the effectiveness of the motivation presented in this article, we removed the frequency domain information modeling section in CRFN so that only the spatial multi-scale feature capture part existed. The implementation results are shown in Table 6. FOTformer generates a performance degradation of 0.55 dB. This means that decomposing the image into the phase and amplitude in the frequency domain and separately learning the potential frequency representation could facilitate clear image reconstruction.

## 5. Discussion

In recent years, significant progress has been made in remote sensing image dehazing based on Transformers. However, the problem of high computational load limits the practical application of these methods. Many efforts have been made to alleviate this problem. Some methods use smaller or fewer tokens to compress the computation range of tokens or utilize the characteristics of low-rank approximation and matrix multiplication to reduce the computational complexity of dot product attention, such as UFormer [33]. In contrast to the aforementioned work of performing feature aggregation in space, some methods choose to perform matrix multiplication in the channel direction to evaluate the self-similarity of features, such as RSDformer [21]. Although these methods are effective at improving computational efficiency, they sacrifice the model’s learning ability, making it unable to effectively capture global features and leading to significant texture loss in the restored image, especially when processing high-resolution images. The results in Table 1 and Figure 3, Figure 4 and Figure 5 also reveal this point, where it can be observed that compared with UFormer and RSDformer, FOTformer generated cleaner background and reliable texture, avoiding obvious fog effect residues and blurred content. Thanks to the frequency-prompt attention evaluator, the FOTformer can effectively learn background details and process the interference of remote sensing haze, achieving the separation of background content and haze degradation. In addition, we also noticed that some works also shifted their attention to the frequency domain to improve the computational efficiency, such as DCTformer [48]. DCTformer adopts matrix multiplication and softmax operators to perform feature autocorrelation evaluation. In fact, matrix multiplication and softmax operators are the main reasons for the high computational load. Thus, DCTformer still has a significant computational cost requirement, with a computational complexity of $O({\bar{N}}^{2}C)$, where *N* is the length of the input sequence and *C* represents the channel numbers of the feature. It is worth noting that the proposed method exploits the element-wise Hadamard product in the frequency domain instead of matrix multiplication in the spatial domain to model global contextual information, which implicitly increases the receptive field of the model and helps to achieve high-quality image reconstruction. According to the properties of FFT, the proposed attention computation complexity is O(NClogN), which is smaller than DCTformer. Moreover, due to the conjugate symmetry of FFT, this study only used the real part of FFT, which further reduced the complexity. Although FOTformer achieved promising remote sensing dehazing results, there are still some limitations. Since the proposed method is based on fully supervised learning methods for single datasets with different haze levels, the generalization of the model is still insufficient. The reason behind the failure of this case is the catastrophic forgetting problem of the model. Hence, potential optimization directions are as follows: (1) Introduce contrastive learning to learn the distribution of different haze concentrations. (2) Use unsupervised learning or combine with pre-trained models to enhance the model’s generalization ability.

## 6. Conclusions

This paper presents a frequency-oriented remote sensing image-dehazing Transformer to explore information in the frequency domain to eliminate disturbances caused by haze in remote sensing images. This method first adopts prompt components to mine rich feature relationships in images and transform these features into the frequency domain. Then, the feature self-correlation is evaluated using element-wise products instead of traditional matrix multiplication in the spatial domain to achieve efficient feature fusion for haze removal. Next, different components in the frequency domain are explored and the global frequency domain information is integrated for reconstructed global contents. Furthermore, information between the frequency and spatial domains are combined and aggregated to promote the propagation of features from the encoder stream to the decoder, reducing information loss. The efficacy of the proposed method was demonstrated through extensive experiments conducted on synthetic and real remote sensing image hazy datasets. In future work, we plan to introduce contrastive learning that is unsupervised or combined with pre-trained models to learn the distribution of different haze concentrations to enhance the model’s generalization ability. 

## Figures and Tables

**Table 3:** Comparison of model efficiency with other methods on a 256×256 image.

Methods	PFFNet	FFA-Net	MSBDN	SCANet	DehazeFormer	UFormer	AIDNet	RSDformer	Ours
Parameters (M)	20.85	4.45	31.35	2.38	9.68	20.60	27.12	4.27	**8.56**
FLOPs (G)	7.24	287.53	41.52	17.65	89.85	41.09	439.92	50.12	**83.19**

**Table 4:** Ablation studies for different components in the model.

Model	FPAE	CRFN	SFAB	PSNR	SSIM	LPIPS
Baseline				23.46	0.9044	0.0667
(a)	✓			25.08	0.9134	0.0648
(b)		✓		24.60	0.9080	0.0705
(c)	✓	✓		24.44	0.9137	0.0617
(d)	✓		✓	24.64	0.9142	0.0627
(e)		✓	✓	24.99	0.9101	0.0563
(f)	✓	✓	✓	**25.32**	**0.9153**	**0.0619**

**Table 5:** Ablation studies for different loss functions in the model.

Model	$L_{1}$	$L_{\mathrm{fft}}$	$L_{\mathrm{edge}}$	PSNR	SSIM	LPIPS
(g)	✓			24.83	0.9123	0.0687
(h)	✓	✓		25.16	0.9141	0.0705
(i)		✓	✓	24.43	0.9141	0.0568
(j)	✓	✓	✓	25.32	0.9153	0.0619

**Table 6:** Ablation studies for designs of the FPAE and CRFN.

Model	w/o LPB	w/o FCR	PSNR	SSIM	LPIPS
(k)	✓		24.64	0.9127	0.0538
(l)		✓	24.77	0.9145	0.0552
(m)	✓	✓	25.32	0.9153	0.0619

## Data Availability

The online experimental datasets used in this study are available at https://www.dropbox.com/s/k2i3p7puuwl2g59/Haze1k.zip?dl=0 (accessed on 16 May 2024).
